# Dietary Patterns Characterized by High Meat Consumption Are Associated with Other Unhealthy Life Styles and Depression Symptoms

**DOI:** 10.3389/fnut.2017.00025

**Published:** 2017-06-14

**Authors:** Maria João Gregório, Ana M. Rodrigues, Mónica Eusébio, Rute Dinis Sousa, Sara Dias, Beate André, Kjersti Grønning, Pedro S. Coelho, Jorge M. Mendes, Pedro Graça, Geir A. Espnes, Jaime C. Branco, Helena Canhão

**Affiliations:** ^1^EpiDoC Unit, Centro de Estudos de Doenças Crónicas (CEDOC) da NOVA Medical School, Universidade Nova de Lisboa (NMS/UNL), Lisboa, Portugal; ^2^Escola Superior de Saúde do Instituto Politécnico de Leiria, Leiria, Portugal; ^3^Faculdade de Ciências da Nutrição e Alimentação da Universidade do Porto, Porto, Portugal; ^4^Programa Nacional para a Promoção da Alimentação Saudável, Direção-Geral da Saúde, Lisboa, Portugal; ^5^Sociedade Portuguesa de Reumatologia, Lisboa, Portugal; ^6^Rheumatology Research Unit, Instituto de Medicina Molecular, Lisboa, Portugal; ^7^Department of Public Health and Nursing, Faculty of Medicine and Health Sciences, Norwegian University of Science and Technology, Trondheim, Norway; ^8^NTNU Center for Health Promotion Research, Trondheim, Norway; ^9^NOVA Information Management School, Universidade Nova de Lisboa, Lisboa, Portugal; ^10^Serviço de Reumatologia do Hospital Egas Moniz – Centro Hospitalar Lisboa Ocidental (CHLO – E.P.E.), Lisboa, Portugal

**Keywords:** dietary patterns, lifestyles behaviors, health status, mental health, Portugal

## Abstract

**Objective:**

We aimed to identify dietary patterns (DPs) of Portuguese adults, to assess their socioeconomic, demographic, lifestyle determinants, and to identify their impact on health.

**Design:**

EpiDoC 2 study included 10,153 Portuguese adults from the EpiDoC Cohort, a population-based study. In this study, trained research assistants using computer-assisted telephone interview collected socioeconomic, demographic, dietary, lifestyles, and health information from March 2013 to July 2015. Cluster analysis was performed, based on questions regarding the number of meals, weekly frequency of soup consumption, vegetables, fruit, meat, fish, dairy products, and daily water intake. Factors associated with DP were identified through logistic regression models.

**Results:**

Two DPs were identified: the “meat dietary pattern” and the “fruit & vegetables dietary pattern.” After multivariable adjustment, women (OR = 0.52; *p* < 0.001), older adults (OR = 0.97; *p* < 0.001), and individuals with more years of education (OR = 0.96; *p* = 0.025) were less likely to adopt the “meat dietary pattern,” while individuals in a situation of job insecurity/unemployment (OR = 1.49; *p* = 0.013), *Azores* island residents (OR = 1.40; *p* = 0.026), current smoking (OR = 1.58; *p* = 0.001), daily alcohol intake (OR = 1.46; *p* = 0.023), and physically inactive (OR = 1.86; *p* < 0.001) were positively and significantly associated with “meat dietary pattern.” Moreover, individuals with depression symptoms (OR = 1.50; *p* = 0.018) and the ones who did lower number of medical appointments in the previous year (OR = 0.98; *p* = 0.025) were less likely to report this DP.

**Conclusion:**

Our results suggest that unhealthy DPs (meat DP) are part of a lifestyle behavior that includes physical inactivity, smoking habits, and alcohol consumption. Moreover, depression symptoms are also associated with unhealthy DPs.

## Introduction

Dietary factors and other lifestyles such as physical inactivity, tobacco use, and harmful ingestion of alcohol are well documented risk factors for several non-communicable chronic diseases (NCDs) and death (1). In fact, an inverse association of healthful dietary patterns (DPs) with all-cause mortality, cancer and cardiovascular disease risk was reported in several studies (2–6). Taking into account these trends in global health, World Health Organization (WHO) has established the prevention and control of NCDs as a major public health challenge, suggesting the need to strengthen the actions to reduce the modifiable risk factors (7). In Portugal, the leading risk factors for disease burden, measured in disability-adjusted life years (DALYs), are unhealthy dietary habits, accounting for 19.2% of all DALYs (8). In terms of dietary behaviors, an excessive consumption of energy, saturated fats, trans fats, sugar and salt and low consumption of vegetables, fruits, and whole grains are pointed to as the most important factors related to these leading causes of death, disease, and disability (9–14).

Improving populations’ health requires epidemiological information on peoples’ health and lifestyles to further develop and target suitable interventions to population groups at risk. Indeed, the inequality gap in NCDs and their risk factors highlight the need of studying the DPs, as well as, their association with socioeconomic and demographic factors, lifestyle behaviors, and health status of the population. Several studies have pointed out the existence of a social gradient in dietary habits, showing that inadequate DPs are more prevalent among individuals from lower socioeconomic status. Educational level, income, and occupation have been suggested as the main socioeconomic factors associated with dietary habits (15). Moreover, a large body of epidemiologic data showed that inadequate dietary habits commonly coexist with other unhealthy lifestyle behaviors (physical inactivity, smoking habits, and alcoholic habits) in the same groups of the population (16–22).

In fact, in Portugal, the lack of epidemiological information, valid and useful to support public health decision-making, mainly in terms of food consumption and its association with NCDs data is a reality. The major national population health surveys (health interview survey, serological survey, and food and nutrition survey) have been conducted on irregular basis and there are no recent data. There is an urgent need to obtain information regarding health-related behaviors of the Portuguese population, providing information related to its determinants (socioeconomic and demographic factors), associated factors, and its consequences for health. Interestingly, one of the aims of Portuguese National Program for the Promotion of Healthy Eating (23) is to collect updated data on food consumption and nutrition, mainly among vulnerable population to take actions toward better health indicators.

Therefore, studying the determinants of DPs is of utmost importance when developing public health policies, identifying population groups that most likely would benefit from those interventions (24). Indeed, the considerably high attributable risk of NCDs due to lifestyles highlights the need of studying the role of DPs in populations with different exposure ranges, according to socioeconomic and demographic factors. It is also important to determine the associations between DPs and other behavioral and clinical variables of interest, in order to better identify vulnerable populations (25).

This study aims to identify DP of Portuguese adults, to assess their socioeconomic, demographic, and lifestyle determinants, and to identify their impact on health.

## Materials and Methods

### The Portuguese Setting

**Figure 1:**
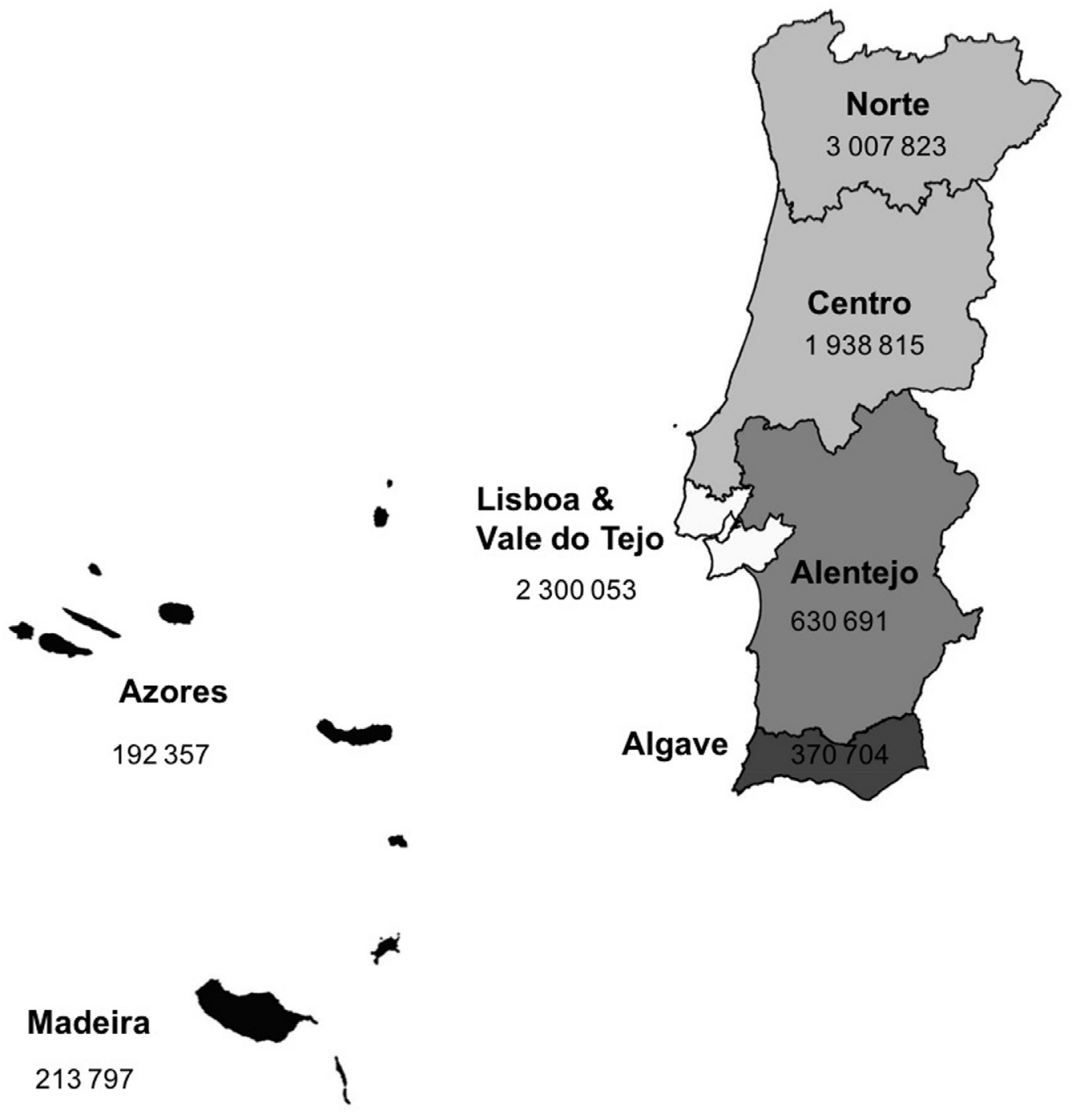
Portugal population density distribution.

Portugal is a South-western European country. According to 2011 Census, the Portuguese resident population was 10,562,178 inhabitants, 8,000,000 out of them are adults. According to Nomenclature of Territorial Unit for Statistics (NUTS II), Portugal is divided into the following seven regions: Norte, Centro, Lisboa e Vale do Tejo, Alentejo, Algarve, Azores, and Madeira (Figure 1).

### Study Design and Participants

A random sample representative of the adult Portuguese population was recruited to constitute the EpiDoC cohort. EpiDoC cohort was designed to study health determinants and outcomes, NCDs, and their impact on health resources consumption. In each evaluation, a nuclear questionnaire regarding socioeconomic, chronic diseases in particular rheumatic diseases, quality of life, and health consumption was applied and was repeated at every evaluation in order to gather longitudinal data. Moreover, each wave of evaluation had also specific and distinct questions regarding other several health and health-related issues that allow obtaining cross-sectional and longitudinal data from these population-based studies. At the first study (EpiDoC 1 or EpiReumaPt), the main goal was to determine the prevalence of rheumatic diseases and their burden in Portugal (26, 27). In EpiDoC 2, the main goal was to characterize lifestyles, health innovation, and social interactions.

**Figure 2:**
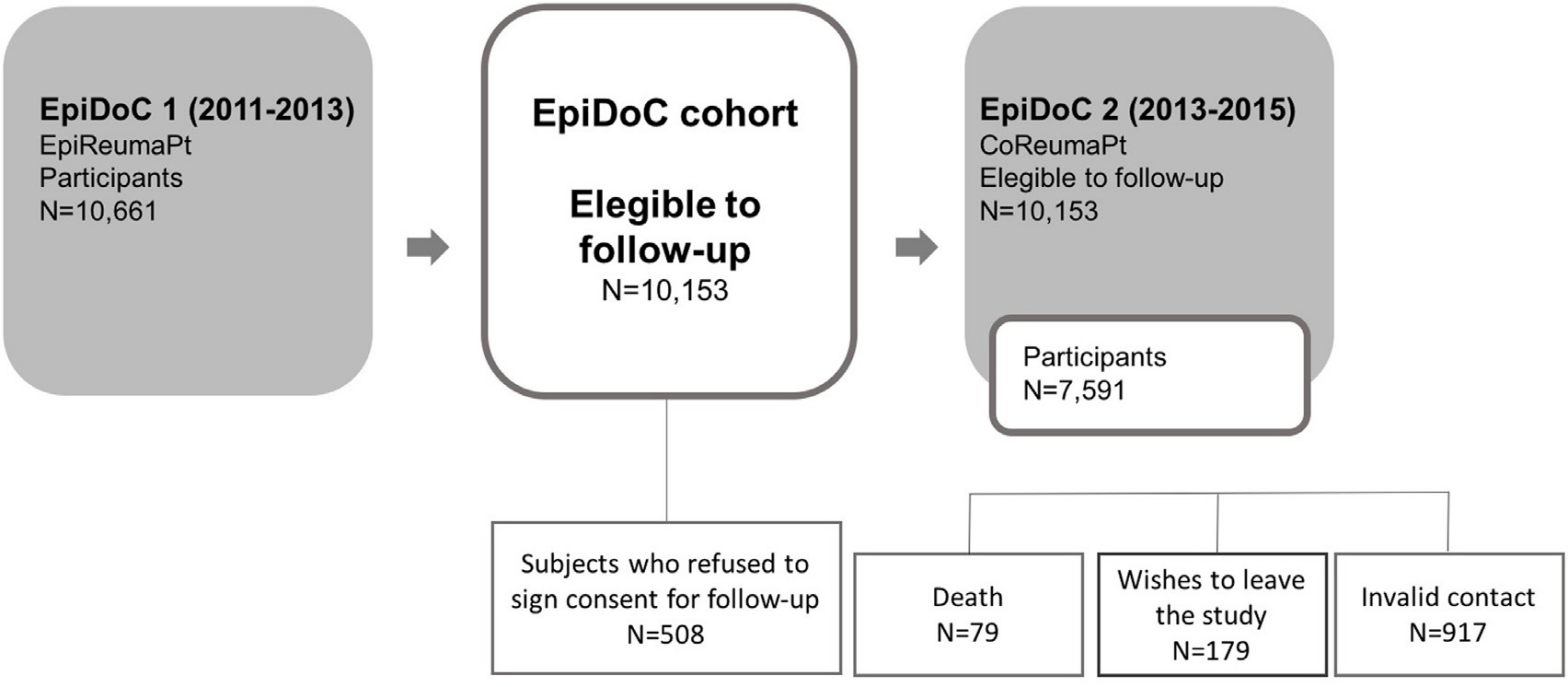
Flowchart of EpiDoc study design.

All the participants in EpiDoC 1 assessment who signed the Informed Consent for further evaluations and those who provided their telephone number were enrolled in the cohort. Subjects unwilling to sign the Informed Consent, unable to speak Portuguese, or with an inability to answer the questionnaire were excluded (28). Regardless, a caregiver might have been the one answering the questionnaire. The flowchart of EpiDoC cohort is described in Figure 2.

### Data Collection

The data collection was performed between March 26, 2013 and July 27, 2015. A trained research assistant team was responsible for collecting the data from these subjects, by telephone calling all the individuals. When a contact was not available, they attempted different moments (morning, afternoon, evening, and weekends) in order to accomplish a minimum of six attempts. The last contact had at least a 1-month interval from the previous one. Only then the contact is abandoned. Rescheduling of the phone interviews was an option.

The interview was telephonically performed with the assistance of a computer-assisted telephone interview system (an in-house software platform, developed by the informatics team of *Sociedade Portuguesa de Reumatologia*). Standardized procedures were undertaken to collect data.

### Measurements

#### Sociodemographic, Characteristics

Sociodemographic (sex, age, ethnicity, years of education, marital status, household composition, and NUTS II) as well as socioeconomic variables (household income, employment status) were collected in EpiDoC 1 [EpiReumaPt study (26)]. At this second assessment (EpiDoC 2), subjects were asked whether there have been changes in those variables.

#### Health Characteristics

During EpiDoC 2 study, individuals were asked if they had been previously diagnosed with some chronic disease (high cholesterol level, high blood pressure, rheumatic disease, allergy, gastrointestinal disease, mental disease, cardiac disease, diabetes, thyroid and parathyroid disease, urolithiasis, pulmonary disease, hyperuricemia, cancer, neurologic disease, and hypogonadism). Health-related quality of life was assessed using the European Quality of Life questionnaire with five dimensions and three levels (EQ-5D-3L) (29, 30), where higher scores correspond to higher quality of life. Physical function was evaluated based on the Health Assessment Questionnaire (0–3, the higher the worse functional ability) (31). The Hospital Anxiety and Depression Scale (32) was applied to identify symptoms of anxiety and depression (score > 11). All these assessment scales were Portuguese-validated versions.

#### Lifestyle Characteristics

Self-reported height and weight were collected during EpiDoC 1 assessment. Body mass index (BMI, weight/height^2^, in kg/m^2^) was calculated and categorized according to the WHO classification into four categories: underweight (BMI < 18.5 kg/m^2^), normal (BMI between 18.5 and 24.9 kg/m^2^), overweight (BMI between 25 and 29.9 kg/m^2^) and obesity (BMI ≥ 30 kg/m^2^) (33).

During EpiDoC 2 interview (2013–2015), questions concerning lifestyle habits were collected and included frequency of alcohol intake (*daily, occasionally, never*), quantity of alcohol units per week (*less than or equal than three alcohol units per week; more than three alcohol units per week but less than three alcohol units per day; more than three alcohol units per day*); smoking habits (*daily, occasionally, past smoker, never smoked*); frequency and type of physical activity; sleep habits (number of hours of sleep per day, categorized in <*6* and ≥*6 h/day*); frequency of watching TV (categorized in *does not watch*, ≤*2 h/day, 3–4 h/day*, and ≥*5 h/day*) and frequency of use of computer/videogames/tablets (categorized in *does not use*, ≤*2 h/day, 3–4 h/day*, and ≥*5 h/day*). Physical activity level was classified based on the question related to the reported weekly frequency of physical activity, and three different categories of physical activity level were obtained according to the following criteria: *inactive* (<1h/week), *moderately active* (between 1 and 2.5 h/week), and *active* (≥2.5 h/week).

Dietary intake was assessed through a group of questions, namely food frequency questions for the following foods and beverages: soup^1^, vegetables, fresh fruit, milk and other dairy products (Question: “How many times per week do you eat or drink…?” Categories of response: *everyday, 6 times/week, 3–5 times/week, 1–2 times/week, rarely*, and *never*), meat, and fish (Question: “How many meals of … do you consume per week?” Categories of response: *10–14 meals/week, 7–10 meals/week, 4–6 meals/week, 1–3 meals/week, rarely*, and *never*). Questions regarding the dietary intake also included the number of meals per day (2, 3, 4, 5, or more meals per day) and the amount of water daily consumed (*1–2, 3–4, 5–7*, >*7 glasses of 150 mL/day*).

### Statistical Analysis

In order to guarantee the representativeness of the sample in relation to the Portuguese population (Mainland and *Madeira* and *Azores* islands), extrapolation weights were computed and used in further statistical analysis. These were obtained by calibrating the extrapolation weights originally designed for the EpiDoC 1 (EpiReumaPt) sample. We first compared the participants and non-participants of the EpiDoC 2 study, concerning their sociodemographic, socioeconomic, and health status characteristics. Based on this comparison, we adjusted the weights based on the stratification by NUT II region, sex, age group (resulting from the aggregation of the original classes in 18–35, 36–55, 56–65, ≥66 years in the *Norte, Centro*, and *Lisboa* regions, and 18–65, ≥66 years in *Alentejo, Algarve*, the *Azores*, and *Madeira*) (26).

Individuals were classified according to their DPs and two clusters were built based on dietary intake (number of meals, weekly frequency of consumption of soup, vegetables, fresh fruit, meat, fish, milk/dairy products, and water intake per day). The scales were very different among questions which implied standardization procedures by rescaling the variables to have a mean of zero and a SD of 1. For the cluster analysis, Ward’s Linkage (34) was used with squared Euclidian distance (35). Of note, food pattern studies are already widely used in Nutritional Epidemiology, and Cluster analysis is one of the methods commonly adopted (36–39).

Since the original questions were recorded as categorical variables, a conversion was made with the purpose of obtaining continuous variables, appropriate for this analysis. A weekly consumption measure was created for each question, considering the middle point for each category.

For the number of meals, it was considered that the common maximum daily number of meals was 6, and therefore, the last category (5 or more meals/day) was recoded using the middle point of 5.5 meals times per day for the 7 days of the week. Thus, the original categories of 2 meals/day, 3 meals/day, 4 meals/day and 5 or more meals/day were recoded, respectively, in the following weekly frequency 14, 21, 28, and 38.5.

For the soup, vegetables, fruit, and milk/dairy products frequency of consumption, it was assumed that everyday consumption can range from once to twice a day, translating in 7–14 times a week, which results in a middle point of 10.5 times a week. For the rarely category, it was reasonable to consider a monthly frequency of consumption, being recoded using 0.25 (once a month—1 time in 4 weeks). Thus for these questions, the original categories of everyday, 6 times/week, 3–4 times/week, 1–2 times/week, rarely, and never were recoded, respectively, in the following weekly frequency 10.5, 6, 4, 1.5, 0.25, and 0.

For the frequency of consumption of meals of meat and fish, it was reasonable to consider a twice monthly based frequency of consumption for the rarely category, since for meat and fish, the question was related to the number of meals per week instead of number of times per week. The original categories of 10–14 meals/week, 7–10 meals/week, 4–6 meals/week, 1–3 meals/week rarely, and never were recoded, respectively, in the following weekly frequency 12, 8.5, 5, 2, 0.5, and 0.

For the highest category of water intake, it was considered the standard recommendation of up to 2 L per day, which translates into up to 14 glasses of 150 mL (approximately 2 L), being the middle point 10.5 glasses per day, which correspond to the middle point of 73.5 glasses per week. The original categories of 1–2 glasses/day, 3–4 glasses/day, 5–7 glasses/day, and >7 glasses/day were recoded, respectively, in the following weekly frequency 10.5, 24.5, 42, and 73.5 mL.

Absolute frequencies and weighted proportions were used to summarize categorical variables. Continuous variables were described by weighted mean values and SDs.

Logistic regression models were used to assess the differences between the obtained clusters of DPs, regarding sociodemographic, socioeconomic, health status, and lifestyle variables. These models were always adjusted for sex, age, and NUT II. The associations between DPs and sociodemographic, socioeconomic, health status, and lifestyle variables were further investigated based on crude and adjusted (age, sex, education, employment status, NUT II, smoking habits, physical activity, and alcohol habits) models. Multivariable models were performed using backward selection. Results were described in terms of odds ratio (OR) and corresponding 95% confidence intervals. Significance level was set at 0.05. Possible interactions between variables introduced in the model that might be correlated were tested.

All analyses were weighted and performed using Stata IC version 12 (StataCorp. 2011. Stata Statistical Software: Release 12; StataCorp LP, College Station, TX, USA).

### Ethics

The study of the subjects was performed according to the principles established by the Declaration of Helsinki (40) and revised in 2013 in Fortaleza. The study was reviewed and approved by the National Committee for Data Protection (*Comissão Nacional de Proteção de Dados*) and by the NOVA Medical School Ethics Committee. The participants provided written informed consent to contribute in all phases of the study.

## Results

In EpiDoC 2 study, a total of 7,591 participants completed the questionnaire. The EpiDoC cohort’s sociodemographic characteristics did not differ from the Portuguese population (Table 1). Table 2 presents the dietary habits description of EpiDoC population.

**Table 1:** Sociodemographic characteristics of the adult Portuguese population: EpiDoC 2 and Census 2011 (41) populations (Portuguese population).

	EpiDoC 2, *n* = 7,591	Census 2011, *n* = 8,657,240
**Sex**		
Female	4,784 (52.2%)	4,585,118 (53.0%)
**Age** (mean ± SD)	48.02 (18.02)	41.31 (16.28)
**Age group**		
18–29	621 (18.4%)	1,470,782 (17.0%)
30–39	975 (18.7%)	1,598,250 (18.5%)
40–49	1,437 (18.2%)	1,543,392 (17.8%)
50–59	1,437 (16.2%)	1,400,011 (16.2%)
60–69	1,440 (13.2%)	1,186,442 (13.7%)
70–74	645 (6.2%)	496, 438 (5.7%)
≥75	1,036 (9.1%)	961, 925 (11.1%)
**Ethnicity/race**		
Caucasian	7,423 (97.1%)	No comparable data
Black	119 (2.5%)	
Asian	3 (0.0%)	
Gipsy	7 (0.1%)	
Other	22 (0.3%)	
**Years of education** (mean ± SD)	8.66 (3.90)	No comparable data
**Education level**		
>12 years	1,336 (22.2%)	1,741,567 (20.1%)
10–12 years	1,391 (24.8%)	1,560,958 (18.0%)
5–9 years	1,547 (21.3%)	2,134,401 (24.6%)
0–4 years	3,272 (31.7%)	3,239,724 (37.4%)
**Nomenclature of Territorial Unit for Statistics**		
*Norte*	2,240 (35.8%)	3,007,823 (34.7%)
*Centro*	1,504 (23.3%)	1,938,815 (22.4%)
*Lisboa*	1,588 (25.4%)	2,300,053 (26.6%)
*Alentejo*	422 (7.2%)	633,691 (7.3%)
*Algarve*	245 (3.8%)	370,704 (4.3%)
*Azores*	793 (2.1%)	192,357 (2.2%)
*Madeira*	799 (2.4%)	213,797 (2.5%)
**Marital status**		
Single	1,285 (28.4%)	No comparable data
Married	4,591 (53.2%)	
Divorced	556 (6.8%)	
Widow(er)	910 (7.3%)	
Consensual union	244 (4.2%)	
**Household income**		
<500€	1,331 (18.0%)	No comparable data
501–750€	1,257 (20.8%)	
751–1,000€	943 (19.0%)	
1,001–1,500€	852 (17.5%)	
1,501–2,000€	511 (10.9%)	
2,001€–2,500€	295 (5.7%)	
2,501–3,000€	188 (3.8%)	
3,001–4,000€	108 (2.1%)	
>4,000€	73 (2.2%)	
**Household composition** (mean ± SD)	2.85 (1.24)	No comparable data
**Household composition**		
1 person	1,121 (11.9%)	No comparable data
2 people	2,710 (32.1%)	
3 people	1,901 (27.9%)	
≥4 people	1,859 (28.2%)	
**Single-parent family**	236 (2.9%)	No comparable data
**Employment status**		
Employed full-time	3,167 (52.7%)	No comparable data
Employed part-time	224 (3.3%)	
Domestic worker	643 (4.7%)	
Unemployed	613 (9.3%)	
Student	149 (4.1%)	
Temporally work disabled	145 (1.6%)	
Retired	2,438 (24.2%)	

*Sample size is not constant due to missing data*.

*EpiDoc—ethnicity/race (*n* = 7,574), years of education (*n* = 7,573), education level (*n* = 7,546), marital status (*n* = 7,586), household income (*n* = 5,558), and employment status (*n* = 7,379)*.

*Female—ethnicity/race (*n* = 4,776), years of education (*n* = 4,774), education level (*n* = 4,759), marital status (*n* = 4,781), household income (*n* = 3,514), employment status (*n* = 4,661)*.

*Male—ethnicity/race (*n* = 2,798), years of education (*n* = 2,799), education level (*n* = 2,787), marital status (*n* = 2,805), household income (*n* = 2,044), employment status (*n* = 2,718)*.

*18–64 years—ethnicity/race (*n* = 5,185), years of education (*n* = 5,189), education level (*n* = 5,184), marital status (*n* = 5,195), household income (*n* = 3,779), and employment status (*n* = 5,103)*.

* ≥ 65 years—ethnicity/race (*n* = 2,389), years of education (*n* = 2,384), education level (*n* = 2,362), marital status (*n* = 2,391), household income (*n* = 1,779), and employment status (*n* = 2,276)*.

**Table 2:** Description of dietary habits of the adult Portuguese population: EpiDoC 2.

	EpiDoC 2, *n* = 7,591
**Number of meals**	
2 meals/day	341 (5.5%)
3 meals/day	2,239 (33.7%)
4 meals/day	1,921 (33.5%)
5 or more meals/day	1,566 (27.2%)
**Frequency of soup consumption**	
Everyday	2,581 (39.8%)
6 times/week	247 (3.0%)
3–5 times/week	1,540 (24.0%)
1–2 times/week	965 (17.7%)
Rarely	573 (12.0%)
Never	169 (3.4%)
**Frequency of vegetables consumption**	
Everyday	3,281 (53.0%)
6 times/week	396 (5.5%)
3–5 times/week	1,559 (26.2%)
1–2 times/week	570 (10.4%)
Rarely	197 (3.5%)
Never	69 (1.4%)
**Frequency of fresh fruit consumption**	
Everyday	4,697 (76.1%)
6 times/week	160 (1.9%)
3–5 times/week	705 (12.7%)
1–2 times/week	295 (5.5%)
Rarely	165 (3.0%)
Never	46 (0.9%)
**Frequency of meat consumption**	
10–14 meals/week	1,022 (20.2%)
7–10 meals/week	1,820 (31.5%)
4–6 meals/week	1,683 (29.0%)
1–3 meals/week	1,367 (17.0%)
Rarely	107 (1.2%)
Never	51 (1.0%)
**Frequency of fish consumption**	
10–14 meals/week	206 (2.7%)
7–10 meals/week	1,296 (20.7%)
4–6 meals/week	1,812 (29.8%)
1–3 meals/week	2,485 (43.5%)
Rarely	161 (2.4%)
Never	46 (0.9%)
**Frequency of milk/diary products consumption**	
Everyday	4,553 (74.6%)
6 times/week	103 (2.0%)
3–5 times/week	575 (9.9%)
1–2 times/week	333 (5.0%)
Rarely	233 (4.1%)
Never	266 (4.5%)
**Daily water intake**	
1–2 glasses/day	755 (10.7%)
3–4 glasses/day	1,522 (23.5%)
5–7 glasses/day	2,038 (35.1%)
>7 glasses/day	1,725 (30.8%)

*Sample size is not constant due to missing data*.

*EpiDoc—number of meals (*n* = 6,067), frequency of soup consumption (*n* = 6,075), frequency of vegetables consumption (*n* = 6,072), frequency of fresh fruit consumption (*n* = 6,068), frequency of meat consumption (*n* = 6,050), frequency of fish consumption (*n* = 6,006), frequency of milk/diary products consumption (*n* = 6,063), and daily water intake (*n* = 6,040)*.

### Dietary Patterns

According to the study purpose, two clusters were identified: cluster 1 with 1,312 individuals and cluster 2 with 4,642 individuals. Cluster 1 was classified as “unhealthier” DP than cluster 2, considering the means of the standardized variables for weekly consumption for each cluster (Table 3). The comparison of such means by clusters allows us to observe that there is a clear difference between the groups, regarding the pattern of food consumption. Cluster 1 (“meat dietary pattern”) has a reduced number of meals per week, less frequency of consumption of soup, vegetables, fresh fruit, fish, milk/dairy products, and less water intake. Cluster 1 has the higher frequency of consumption of meat. Cluster 2 (“Fruit & vegetables dietary pattern”) has a higher number of meals per week, high frequency of consumption of soup, vegetables, fresh fruit, fish, milk/dairy products, and high water intake. Cluster 2 has a lower frequency of consumption of meat.

**Table 3:** Mean values of the padronized variables for weekly consumption for each cluster and for the entire population.

	“Meat dietary pattern”	“Fruit & vegetables dietary pattern”	Total
Number of meals	−0.3077	0.0881	0.0009
Soup consumption	−0.3179	0.0901	0.0002
Vegetables consumption	−0.4800	0.1347	−0.0007
Fresh fruit consumption	−1.7850	0.5024	−0.0016
Meat consumption	0.1531	−0.0344	0.0069
Fish consumption	−0.2877	0.0789	−0.0019
Dairy products consumption	−0.1839	0.0583	0.0050
Water intake	−0.1331	0.0385	0.0007

### Sociodemographic and Lifestyle Characteristics Associated with “Meat Dietary Pattern”

We performed logistic regression models in order to identify the associated factors with the “unhealthy” DP—“meat dietary pattern” (Tables 4 and 5). We found that women compared to men (OR = 0.52; *p* < 0.001), people of higher age (OR = 0.97; *p* < 0.001), and people with more years of education (OR = 0.96; *p* = 0.025) are less likely to adopt the meat DP. On the other hand, individuals in a situation of unemployment/part-time employment/domestic worker (OR = 1.49; *p* = 0.013) were more likely to adopt this DP than their counterparts (employed full-time/student/temporally work disabled/retired). Individuals from *Azores* tend to be more prone to belong to this group (OR = 1.40; *p* = 0.026 vs *Norte*) (Table 4).

**Table 4:** Crude and adjusted odds ratio (OR) for the association between sociodemographic and lifestyle characteristics of “meat dietary pattern.”

	“Meat dietary pattern”	“Fruit & vegetables dietary pattern”	Crude OR (95% confidence interval, CI)	*p*-Value	Adjusted OR (95% CI)	*p*-Value
**SOCIODEMOGRAPHIC CHARACTERISTICS**						
**Sex**						
Female	646 (37.5%)	2,678 (51.5%)	0.57 (0.46; 0.70)	<0.001^†^	0.52 (0.40; 0.67)	<0.001^†^
**Age**	41.31 (16.28)	48.30 (17.99)	0.98 (0.97; 0.98)	<0.001^†^	0.97 (0.96; 0.98)	<0.001^†^
**Years of education**	9.20 (3.58)	8.78 (3.89)	1.03 (1.00; 1.06)	0.025^†^	0.96 (0.93; 0.99)	0.025^†^
**Employment status**						
Employed full-time/student/temporally work disabled/retired	1,022 (78.94%)	3,850 (84.67%)	1		1	
Unemployed/employed part-time/domestic worker	289 (21.06%)	778 (15.33%)	1.47 (1.09; 1.99)	0.011^†^	1.49 (1.09; 2.03)	0.013^†^
**Marital status**						
Single/divorced/widow(er)	543 (50.6%)	1,566 (40.3%)	1		1	
Married/consensual union	768 (49.4%)	3,071 (59.7%)	0.66 (0.53; 0.82)	<0.001^†^	0.82 (0.65; 1.03)	0.084
**Single-parent family**	55 (3.54%)	141 (2.58%)	1.39 (0.93; 2.07)	0.110	1.54 (0.98; 2.41)	0.060
**NUTII**						
*Norte*	448 (41.0%)	1,497 (37.2%)	1		1	
*Centro*	214 (20.0%)	1,055 (25.0%)	0.73 (0.56; 0.94)	0.013^†^	0.78 (0.60; 1.02)	0.069
*Lisboa*	239 (24.1%)	919 (23.9%)	0.92 (0.66; 1.26)	0.592	0.94 (0.66; 1.34)	0.742
*Alentejo*	44 (5.5%)	234 (6.6%)	0.76 (0.50; 1.15)	0.191	0.77 (0.49; 1.21)	0.256
*Algarve*	33 (3.4%)	136 (3.6%)	0.45 (0.52; 1.38)	0.507	0.79 (0.47; 1.34)	0.380
*Azores*	172 (3.0%)	372 (1.7%)	1.65 (1.25; 2.18)	< 0.001^†^	1.40 [(1.04; 1.89)]	0.026^†^
*Madeira*	162 (3.1%)	428 (2.1%)	1.36 (1.00; 1.84)	0.048	1.32 (0.96; 1.83)	0.092
**LIFESTYLE CHARACTERISTICS**						
**BMI (kg/m^2^)**						
Underweight/normal weight	567 (51.70%)	1,851 (48.12%)	1		1	
Overweight/obesity	674 (48.30%)	2,577 (51.88%)	0.87 (0.69; 1.08)	0.209	1.07 (0.82; 1.40)	0.603
**Smoking habits**						
Never smoked/past smoker	909 (61.53%)	3,864 (77.84%)	1		1	
Current smoker	403 (38.47%)	772 (22.16%)	2.20 (1.69; 2.85)	< 0.001^†^	1.58 [(1.20; 2.09)]	0.001^†^
**Alcohol intake**						
Never consumed	403 (27.4%)	1,802 (36.2%)	1		1	
Occasionally	527 (45.8%)	1,650 (40.4%)	1.50 (1.16; 1.93)	0.002^†^	1.17 (0.87; 1.58)	0.299
Daily	473 (34.5%)	1,802 (37.4%)	1.51 (1.14; 1.99)	0.004^†^	1.46 (1.05; 2.02)	0.023^†^
**SEDENTARY BEHAVIORS**						
**Physical activity**						
Moderate/active	401 (34.94%)	2,030 (46.68%)	1		1	
Inactive	897 (65.06%)	2,572 (53.32%)	1.63 (1.29; 2.05)	< 0.001^†^	1.86 (1.45; 2.39)	< 0.001^†^
**Screen time—watching TV**						
Does not watch	51 (7.1%)	122 (3.1%)	1		1	
≤2 hours/day	862 (63.5%)	3,112 (70.1%)	0.39 (0.20; 0.76)	0.005^†^	0.47 (0.21; 1.05)	0.066
3–4 h/day	291 (22.2%)	1,011 (19.9%)	0.48 (0.24; 0.96)	0.038^†^	0.65 (0.29; 1.49)	0.311
≥5 h/day	105 (7.2%)	385 (7.0%)	0.44 (0.18; 1.07)	0.070	0.64 (0.26; 1.59)	0.336
**Screen time—using computer/videogames/tablets**						
Does not use	553 (28.0%)	2,080 (35.0%)	1		1	
≤2 h/day	415 (39.0%)	1,511 (37.2%)	1.31 (1.00; 1.71)	0.046^†^	0.75 (0.57; 0.99)	0.043^†^
3–4 h/day	107 (11.1%)	278 (7.2%)	1.93 (1.25; 2.96)	0.003^†^	1.01 (0.61; 1.68)	0.972
≥5 h/day	232 (21.9%)	763 (20.6%)	1.33 (1.01; 1.75)	0.042^†^	0.81 (0.54; 1.22)	0.314
**Sleep habits**						
<6 h/day	168 (13.7%)	495 (15.5%)	1		1	
≥6 h/day	654 (86.3%)	2,213 (84.5%)	0.87 (0.64; 1.17)	0.354	1.13 (0.81; 1.60)	0.470
**Search for health information**						
Searchers vs non-searchers	208 (20.38%)	1,014 (27.88%)	0.66 (0.48; 0.92)	0.013^†^	0.73 (0.51; 1.03)	0.069

*Employment status was recoded as 0 if employed full-time/student/temporally work disabled/retired and 1 if unemployed/employed part-time/domestic worker; marital status was recoded as 0 if single/divorced/widow(er) and 1 if married/consensual union; household composition was considered as a continuous variable, giving the number of people in the household; body mass index (BMI) was recoded as 0 if underweight/normal weight and 1 if overweight/obesity; smoking habits was recoded as 0 if never smoked/past smoker and 1 if current smoker; physical activity was recoded as 0 if moderate/active and 1 if inactive; sleep habits was recoded as 0 if <6 h/day and 1 if ≥6 h/day; search for health information was recoded as 0 if non-searchers and 1 if searchers*.

*Odds ratio adjusted for age, sex, education, employment status, NUTII, smoking habits, physical activity and alcohol habits*.

*^†^p-Value < 0.05*.

**Table 5:** Crude and adjusted odds ratio (OR) for the association between health characteristics of “meat dietary pattern.”

	“Meat dietary pattern”	“Fruit & vegetables dietary pattern”	Crude OR (95% confidence interval, CI)	*p*-Value	Adjusted OR (95% confidence interval, CI)	*p*-Value
**Health characteristics**						
Number of NDCs (self-reported)	1.18 (1.47)	1.66 (1.84)	0.83 (0.79; 0.88)	<0.001^†^	0.98 (0.91; 1.05)	0.506
Non-communicable chronic diseases (self-reported)						
High blood pressure	319 (14.4%)	1,448 (24.5%)	0.52 (0.43; 0.63)	< 0.001^†^	0.90 (0.72; 1.13)	0.375
Diabetes	101 (4.5%)	540 (9.3%)	0.45 (0.34; 0.61)	< 0.001^†^	0.74 (0.54; 1.00)	0.055
High cholesterol level	340 (17.7%)	1,459 (25.7%)	0.62 (0.51; 0.76)	<0.001^†^	0.93 (0.74; 1.15)	0.490
Neoplastic disease	40 (1.9%)	218 (4.3%)	0.41 (0.26; 0.65)	<0.001^†^	0.56 (0.31; 1.03)	0.062
Quality of life EQ5D score	0.81 (0.25)	0.79 (0.27)	1.52 (1.05; 2.18)	0.025^†^	0.74 (0.47; 1.19)	0.218
Physical function Health Assessment Questionnaire score (0–3)	0.23 (0.45)	0.32 (0.54)	0.71 (0.60; 0.84)	<0.001^†^	1.13 (0.95; 1.36)	0.177
Anxiety symptoms (score ≥ 11)	200 (10.7%)	577 (10.8%)	0.99 (0.76; 1.28)	0.931	1.19 (0.89; 1.61)	0.240
Depression symptoms (score ≥ 11)	135 (6.3%)	398 (6.5%)	0.98 (0.74; 1.29)	0.867	1.50 (1.07; 2.09)	0.018^†^
Was hospitalized since last contact	171 (10.4%)	787 (15.8%)	0.62 (0.47; 0.81)	0.001^†^	0.81 (0.61; 1.08)	0.144
Number of medical appointments since last contact	4.97 (5.40)	6.32 (6.80)	0.96 (0.94; 0.98)	<0.001^†^	0.98 (0.96; 1.00)	0.025^†^

*OR adjusted for age, sex, education, employment status, NUTII, smoking habits, physical activity, and alcohol habits*.

*^†^p-value < 0.05*.

Inherently, some other lifestyle factors were significantly and independently associated with the adoption of the “meat dietary pattern,” individuals who smoke (OR = 1.58; *p* < 0.001), those who drink alcohol daily (OR = 1.46; *p* = 0.023), and those who are physically inactive (OR = 1.86; *p* < 0.001) seem to opt more for this DP. Furthermore, individuals who spend two or less hours per day using computers, videogames, and tablets (OR = 0.75; *p* = 0.043) were less likely to adopt the “meat dietary pattern” (Table 4).

### Health Characteristics Associated With “Meat Dietary Pattern”

Regarding health status factors, we verified that the adoption of “meat dietary pattern” was significantly associated with depression symptoms (OR = 1.50; *p* = 0.018) (Table 5). Considering health-care resource consumption, individuals with a higher number of medical appointments since last contact (OR = 0.98; *p* = 0.025) were less likely to adopt this DP (Table 5).

### Determinants of “Meat Dietary Pattern”

In the end, we have constructed a final model to identify the independent factors that contribute for the adoption of “meat dietary pattern” in Portugal (Table 6). A situation of unemployment/part-time employment/domestic working was an independent determinant for the adoption of “meat dietary pattern” (OR = 0.33; *p* = 0.041). Female sex (OR = 0.52; *p* < 0.001), elderly (OR = 0.97; *p* < 0.001), and higher literacy (OR = 0.96; *p* = 0.040) were considered protective factors for the adoption of “meat dietary pattern.” Regional disparities were also verified, with living on the *Azores* (OR = 1.44; *p* = 0.017) and *Madeira* (OR = 1.39; *p* = 0.046) islands being independent determinants for the adoption of “lower fruit and vegetables intake and higher meat intake” DP. Regarding lifestyle factors, individuals who were current smokers (OR = 1.50; *p* = 0.003) and physically inactive (OR = 1.80; *p* < 0.001) had 50 and 80%, respectively, higher odds of having a “meat dietary pattern.” Finally, we found a negative association between self-reported diabetes and the adoption of a DP with a lower frequency of consumption of soup, vegetables, and fruit and a higher frequency of meat consumption (OR = 0.72; *p* = 0.035), while a positive association was found between depression symptoms (OR = 1.56; *p* = 0.010) and the adoption of this DP (Table 6).

**Table 6:** Final multivariate model of the determinants of “meat dietary pattern.”

	Adjusted odds ratio (95% confidence interval, CI)	*p*-Value
**Sex**		
Female	0.52 (0.41; 0.67)	<0.001^†^
**Age**	0.97 (0.96; 0.98)	<0.001^†^
**Years of education**	0.96 (0.93; 1.00)	0.040^†^
**Employment status**		
Employed full-time/student/temporally work disabled/retired	1	
Unemployed/employed part-time/domestic worker	1.33 (1.01; 1.75)	0.041^†^
**NUTII**		
*Norte*	1	
*Centro*	0.78 (0.60; 1.03)	0.081
*Lisboa*	0.90 (0.64; 1.27)	0.553
*Alentejo*	0.81 (0.52; 1.27)	0.366
*Algarve*	0.81 (0.48; 1.38)	0.444
*Azores*	1.44 (1.07; 1.95)	0.017^†^
*Madeira*	1.39 (1.01; 1.93)	0.046^†^
**Non-communicable chronic diseases (self-reported)**		
Diabetes	0.72 (0.53; 0.98)	0.035^†^
**Depression symptoms (score ≥ 11)**	1.56 (1.11; 2.19)	0.010^†^
**Smoking habits**		
Never smoked/past smoker	1	
Current smoker	1.50 (1.15; 1.96)	0.003^†^
**Physical activity**		
Moderate/active	1	
Inactive	1.80 (1.41; 2.30)	<0.001^†^

*n = 5768, F_(14, 5,754)_ = 17.68, p < 0.0001*.

*Employment status was recoded as 0 if employed full-time/student/temporally work disabled/retired and 1 if unemployed/employed part-time/domestic worker; smoking habits was recoded as 0 if never smoked/past smoker and 1 if current smoker; physical activity was recoded as 0 if moderate/active and 1 if inactive*.

*^†^p-Value < 0.05*.

## Discussion

Using a representative sample of the adult Portuguese population we identified two distinct DPs through cluster analysis, the “meat dietary pattern” and the “fruit & vegetables dietary pattern.” Similar results were also identified in other countries (using *a posteriori* defined DP), with higher scores on fruits, vegetables, and fish and lower scores in meat indicating a healthier DP (42). Our study has showed that the “unhealthy” DP (“meat dietary pattern”) is independently associated with younger age, lower years of education, male sex, unemployment, part-time employment and domestic working. Furthermore, individuals from *Azores* and *Madeira* were more likely to report this kind of DP, given their high frequency of consumption of meat. Regarding lifestyle behaviors, smoking and being physically inactive were significantly and independently associated with having this type of DP.

The Portuguese third National Health Survey was performed 16 years ago. In this study, they also found that individuals with a higher education level reported to consume more frequently fruit, vegetables, milk and fish, and less wine and spirits than their counterparts from less educated groups. However, in contrast to our findings, the Portuguese third National Health Survey suggested that differences according to income were less clear, since there were no significant differences in food consumption between low and high income groups (43). The actual change can be justified by the worsening economic condition in the last 5 years in Portugal.

The inverse association between an unhealthy DP and socioeconomic status, namely with education and employment status, has been described across different studies in different populations (20, 44, 45). In fact, sex and age were also observed to influence DPs. In general, men tend to have a higher frequency of meat consumption and a lower frequency of consumption of fruit and vegetables. Furthermore, the elderly tend to eat more frequently soup, vegetables, fruit, and fish and less frequently meat. These trends were observed in other studies (19, 21, 46, 47). Also, a recent analysis on DPs in the French population revealed that women were more concerned about healthy eating and that younger people were more likely to have unhealthier DPs. The influence of socioeconomic factors in diet was also confirmed in the DPs in France (48).

Geographical differences in dietary habits in Portugal were also observed. The regions of *Madeira* and *Azores* islands have a significant higher proportion of residents that have the unhealthy pattern. A higher prevalence of overweight and obesity was also found in *Azores* region. Geographical differences in DPs are not surprising since dietary habits are largely influenced by cultural, socioeconomic aspects, and local food availability (25, 49).

Furthermore, our study showed that unhealthy DPs, physical inactivity behaviors, smoking, and daily intake of alcohol coexist in the same individuals. These results were also observed in several other countries worldwide where unhealthy diet is combined with other unhealthy lifestyle behaviors such as physical inactivity, smoking and alcoholic habits (16–22). For instance, a recent study conducted in the English adult population showed that 68% of adults had at least two of the four unhealthy behaviors (unhealthy diet, physical inactivity, smoking, and heavy drinking) and that 26% had a combination of three or more of the four unhealthy behaviors (50). The results of an Irish population study are consistent with our findings, in which DPs are related to other lifestyles behaviors (physical activity and smoking) (51). Health promotion interventions are frequently focused on improving a specific lifestyle behavior. The co-occurrence of unhealthy behaviors in the same individuals suggests that health promotion interventions may be more effective if they focus on a multiple behavior approach than on a single behavior (22). In fact, several studies have demonstrated that the combination of diet and physical activity interventions has higher effect on weigh reduction and other health determinants (cholesterol levels and blood pressure) than when were performed alone. Moreover, these combined interventions also have demonstrated effect on smoking cessation. On the other hand, in subjects with alcohol addiction, treating more than one addiction including smoking will lead to a more sustained alcohol free period. These data reinforce the idea that public policies should focus on promoting multiple health behavioral change (52).

In terms of health outcomes, our study revealed that individuals with the “meat dietary pattern” reported depression symptoms more often. In line with our results, several studies have found that people who have an unhealthy diet were more likely to report symptoms of depression (25, 53–57). Indeed, several explanations can be pointed out for this result. There is some evidence suggesting that a healthy DP, with high intake of fruit, vegetables, whole grains, and fish, may reduce the depression risk. On the other hand, there is also evidence from observational studies that unhealthy diets, namely those with high contents of saturated fat and refined carbohydrates are identified as risk factors for depression. Furthermore, depression in elderly is associated with inability to shop for food and prepare meals (58).

We had tested the interactions between: diabetes and female sex; smokers and female sex; smokers and age; physical activity and female sex; physical activity and age; depression symptoms and female sex; and depression symptoms and age. For all the interactions tested we just found a significant association between depression symptoms and age (OR = 1.02; *p* = 0.04), as well as between depression symptoms and female sex (OR = 2.36; *p* = 0.013). These significant interactions showed that the effect of the observed association between depression symptoms and “meat dietary pattern” is higher as age increases and in female individuals. These interactions were tested because several studies show that women and older individuals are more likely to have depression symptoms.

In our study, individuals with self-reported diabetes were less likely to report the “unhealthy” DP. One explanation could be that, since this is a cross-sectional study, it is possible that participants who had been previously diagnosed with diabetes might have modified their diet, which might explain this unexpected association. This finding might also be a result of social desirability bias in self-report data of dietary intake. In fact, several studies found that after a diagnosis of a chronic disease individuals tend to improve health-related behaviors (59–61).

We did not find associations between the “unhealthy dietary pattern” and overweight or obesity. One possible explanation for the absence of association between “unhealthy dietary pattern” and overweight or obesity could be the fact that our models were not adjusted for total energy intake (62) and/or some of the obese individuals are changing habits to decrease weight.

Our study has limitations that should be pointed out. It is important to highlight that the questions we used to assess dietary intake are limited to a few number of food groups. Thus, we are not able to look at these DPs as a whole. Although in our study we did not use the recommended methods to evaluate food consumption, the DPs found in our study are similar to those found in other studies of DPs analysis, namely in terms of food content of fruit, vegetables, and meat (63, 64). To examine the association between food consumption, socioeconomic factors, lifestyle behaviors, and health outcomes we used a DP approach (*a posteriori* defined DPs), which has the advantage to take into account the synergistic effects of the different nutrients and foods. Eating behavior is a complex issue and its evaluation requires the utilization of relative complex methods to assess food consumption. As a matter of fact, WHO suggested that in nutritional epidemiologic studies, population food consumption should be based on eating patterns (42, 65, 66). Besides, according to a study conducted to analyze the validity and reproducibility of *a posteriori* DPs using food consumption data collected by two distinct methods (food frequency questionnaire and 24-h recall), two DPs similar to those that we found in our study were also found: “fruit and vegetables” DP and “meat” DP. This suggests a reasonable reproducibility of the DPs derived from the different method used to assess food consumption (64). Other limitation is the cross-sectional design, where causal associations are unable to be identified. However, to our knowledge, these data are a major contribution for the understanding of the Portuguese dietary habits since there are no updated data regarding food consumption in a national representative population for Portugal. To our knowledge, this is the first study addressing the associations between DPs, socioeconomic status, lifestyle behaviors, and health outcomes, conducted in a large sample representative for the Portuguese population.

In conclusion, this study has identified diet vulnerable strata (male, young age, lower years of education, unemployment, part-time employment, domestic working, and island residents) among Portuguese adults. Moreover, unhealthy DP characterized by a lower frequency of consumption of soup, vegetables, fruit, fish, milk/dairy, less water intake, and a higher frequency of consumption of meat was significantly associated with depression symptoms. Strategies to promote fruit and vegetables consumption [at least 400 g per day in order to achieve WHO recommendations (67)] and assure the adequate intake of dairy products (2–3 portions per day in order to meet Portuguese food guide) and meat (37.5–112.5 g per day in order to meet Portuguese food guide) (68) must be a priority of public health policies.

Finally, unhealthy DPs are associated with other unhealthy lifestyle behaviors, such as physical inactivity, smoking, and alcoholic habits, which reinforces the idea that behavioral changes interventions should target more than one lifestyle domain (eat habits, physical exercise, alcohol, and smoking habits) (69).

Health education and multiple health behavioral change programs should start immediately among Portuguese vulnerable strata in order to improve their health status.

## Ethics Statement

The study of the subjects was performed according to the principles established by the Declaration of Helsinki (40) and revised in 2013 in Fortaleza. The study was reviewed and approved by the National Committee for Data Protection (*Comissão Nacional de Proteção de Dados*) and by the NOVA Medical School Ethics Committee. The participants provided written informed consent to contribute in all phases of the study.

## Author Contributions

MG and AR contributed equally to this work. HC and AR designed the study. ME and AR analyzed the data. MG and AR interpreted the data and wrote the paper. JB, PG, PC, JM, RS, BA, KG, GE, and HC gave scientific support and revised the manuscript. All the authors discussed the results and implications and commented the manuscript at all stages.

## Conflict of Interest Statement

The authors declare that the research was conducted in the absence of any commercial or financial relationships that could be construed as a potential conflict of interest.

## Footnotes

^1^The question regarding the frequency of soup consumption did not specify that we are asking about the consumption of vegetable soup, however, “soups” in Portugal are traditionally prepared with vegetables.
